# Research Advances of Microencapsulation and Its Prospects in the Petroleum Industry

**DOI:** 10.3390/ma10040369

**Published:** 2017-03-31

**Authors:** Miaomiao Hu, Jintang Guo, Yongjin Yu, Lei Cao, Yang Xu

**Affiliations:** 1 School of Chemical Engineering and Technology, Tianjin University, Tianjin 300350, China; m15822890856@163.com (M.H.); yuyongjindri@cnpc.com.cn (Y.Y.); caolei19850129@126.com (L.C.); xuyang199211@163.com (Y.X.); 2 Drilling Research Institute, China National Petroleum Corporation, Beijing 102206, China

**Keywords:** microcapsule, petroleum industry, self-healing, corrosion inhibitor, controllable release

## Abstract

Additives in the petroleum industry have helped form an efficient system in the past few decades. Nowadays, the development of oil and gas has been facing more adverse conditions, and smart response microcapsules with the abilities of self-healing, and delayed and targeted release are introduced to eliminate obstacles for further exploration in the petroleum industry. However, limited information is available, only that of field measurement data, and not mechanism theory and structural innovation data. Thus we propose that the basic type, preparation, as well as mechanism of microcapsules partly depend on other mature fields. In this review, we explore the latest advancements in evaluating microcapsules, such as X-ray computed tomography (XCT), simulation, and modeling. Finally, some novel microencapsulated additives with unparalleled advantages, such as flexibility, efficiency, and energy-conservation are described.

## 1. Introduction

Petroleum has been ‘instrumentalized’ in the high-stake politics and struggles involving transnational and local social forces, contributing to multiple crises [1], which cannot be regarded only as a source of energy or a mere product with high value. However, chemistry researchers are extremely interested in the rapid progress of petroleum that has triggered the birth and prosperity of related chemical additives. With excessive prosperity in the petroleum industry, the development of oil and gas fields has faced more challenges, such as inadequate storage and complicated working environments. A sea of functional structures is designed to fulfill these harsh requirements, most attractively, microcapsule-based molecules with an average diameter as small as 1 μm to several hundred micrometers, which can overcome the disadvantages of single constituents to satisfy the utilization process and promise a rewarding resolution in oil recovery and oil well cementation. In this review, we introduce the basic information on microcapsules, their applications in oilfield chemicals, recent advances in evaluation, and offer our perspective on future research directions. 

## 2. Research Advances in Microencapsulation 

### 2.1. Development of Microcapsule Structure Polymers

Since the appearance of the new concept of “microencapsulation” in the 1930s, which was sought as a cleaner substitute for carbon paper and carbon ribbons [2], this technology has captured intense interest within the scientific community as well as the industrial world, and has made rapid progress in the past few decades because of the tremendous demand for more advanced molecular structures. Microencapsulation is a process in which very tiny droplets or particles of liquid or solid material are surrounded or coated with a continuous film of polymeric material, which in turn isolate them from the external environment, and is mainly used for the purpose of protection, controlled release, and core compatibility [3,4]. A number of unique and superior products with microcapsules have been either available commercially or are in the process of development. In addition, many patents about microencapsulation have been granted for applications in biomedicine, food, self-healing materials [5,6], and the cosmetics industry. Microcapsules have also been applied in oilfield additives, which were expected to create high economic value in the petroleum industry in recent years. However, up to now, all these efforts translated into very few industrial applications. 

Microencapsulation is used to ensure that the crucial core reaches the area of action without becoming unfavorably affected by the environment through which it passes. The principle reasons for microencapsulation can be summarized as follows:

- To protect core materials from adverse environmental effects (pH, temperature, humidity, and other substances)
- To control the active components for delayed (timed) release or long-acting (sustained) release
- To combine two incompatible components for a multifunctional structure

These three principles play a positive role in its application. As an example, in biomedicine research, its excellent isolation protection and controlled release characteristics have attracted attention. Researchers actively explored the application of microencapsulated cell transplantation and microencapsulated drugs in the treatment of diabetes [7], Parkinson’s disease [8], liver failure [9], tumor [10,11], etc. In self-healing materials, Kim [12] prepared a water-treatment membrane that could restore its water flux and particle rejection properties autonomously. To achieve self-healing, a polyurethane shell acts as a protective coating and also controls the release of the isophorone diisocyanate core. In the food industry, microencapsulation of probiotic bacteria, which can be used to enhance viability during processing and are also utilized for targeted delivery in the gastrointestinal tract [13], has achieved remarkable benefits. Additionally, methods adopted from immobilized cell technology were applied for the microencapsulation of probiotics, often optimized towards specific requirements associated with the protection of probiotic cells in food production. In the cosmetics industry, to improve the stability or bioavailability of products [14], this tiny structure is commonly used to avoid incompatibility of substances, reduce odor of active ingredients, and for the protection of effective chemicals prone to oxidation or reaction, for example, vitamins, sun filters, moisturizers, fragrances, can be kept in chemical inert nylon microspheres [15]. The applications mentioned above are just the tip of the iceberg and further exploration is still needed, so that this promising technology will play a role in broader fields.

### 2.2. Types of Microcapsules

Based on the traditional classification criteria, microcapsules can be classified according to size, core material, and geometry [16,17,18], which has been reported in many excellent reviews [19]. Here we classify according to the stimulations the microcapsules receive for release, that is, press-sensitive, thermo-sensitive [20], photo-sensitive [21,22], and concentration gradient sensitive microcapsules. In thermal-sensitive microcapsules, thermally expandable microcapsules are considered to be the most attractive, due to a wide variety of applications [23,24,25], which also makes full use of the formation temperature differences and is promising in the petroleum industry. The membrane of the microcapsules has a certain blocking effect between the environment and internal phase, but when subjected to the corresponding stimulus, the outer sheath tends to dissolve, rupture, or expand, which can reduce its surface density, and then the core materials will release to affect the outer conditions or react with certain substances outside. This classification is related to the stimulation effect on the shell layer, thus give guidance regarding its application.

### 2.3. Formation Principle of Microcapsule-Based Structures

**Figure 1:**
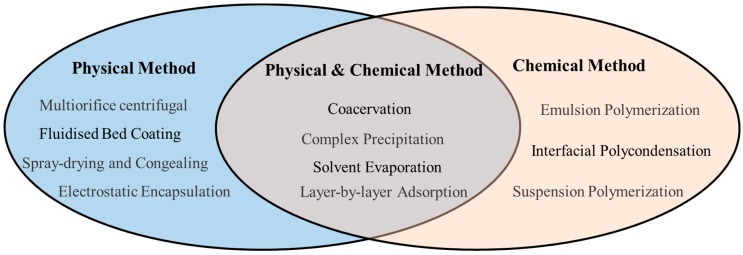
Major microencapsulation methods.

Microencapsulation is a multidisciplinary field, which is regarded as the combination of many branches, such as colloid chemistry, polymer chemistry, physical chemistry, and material science [26,27]. A wide number of methodologies have been reported to fabricate target microcapsules. According to the formation mechanism of the shell material and the indispensable conditions for the preparation of the microcapsules, they can be classified into three branches: chemical methods, physical methods, and physico-chemical methods. Some of these are listed in Figure 1. Physical methods like fluidized bed coating [28] provide an alternative for the fabrication of agents in oil drilling or other related areas, as well as interfacial polymerization as a chemical method and solvent evaporation and coacervation as physico-chemical methods. Fluidized bed coating is one of the few advanced technologies capable of coating particles with basically any type of shell material [29]. This method has the advantage of simple equipment, low cost, convenient operation, and it is also favorable for scale-up [30,31]. However, in terms of scientific research exploration, the other three methods are more appropriate (see detailed introduction below).

#### 2.3.1. Interfacial Polymerization

**Figure 2:**
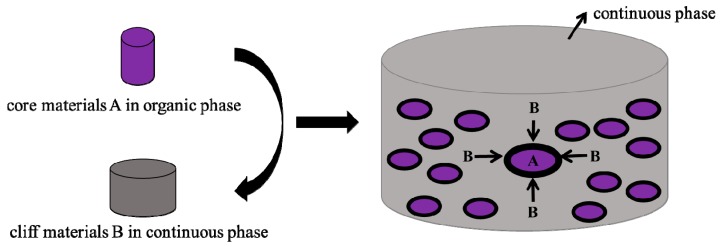
Microencapsulation with interfacial polymerization.

Interfacial polymerization was mainly developed toward the end of the 1960s [32,33], leading to applications in microcapsule production by the mid-1970s [31,32]. A key feature of microencapsulation by interfacial polymerization is the diffusion of the reagents to the reaction interface [34]. In this approach, generally one phase is aqueous and the other consists of an organic solvent. If the aqueous phase is the dispersed phase, the core of the capsules will be hydrophilic, while inverting the phases would lead to a hydrophobic core. This technology allows encapsulating a wide range of core materials, such as aqueous solutions, water-immiscible liquids, and solid particles [35,36]. The mechanism has been shown in Figure 2, where two reactive monomers that are soluble in their respective immiscible phases come into contact at the interface, and then the liquid droplets are enveloped within a polymeric membrane.

Several advanced studies on interfacial polymerization with the ability of direct control of the capsule mean size and membrane thickness have been reported. This property, along with the merits of versatile and stable mechanical and chemical properties of the membrane, as well as permeability, makes this approach a promising candidate for wide applications. Therefore, a multitude of publications have been focused on its utilization, such as self-healing [36,37] agriculture [38], cosmetics [39], and pharmaceutics [40]. Mcilroy and Blaiszik [41] developed a method for the preparation of microcapsules containing a reactive amine with potential applications in self-healing polymers. In this case, the stable control of the specific diameters and membrane thickness via interfacial polymerization makes it possible to optimize the rupture of microcapsules and improve the efficiency of the self-healing materials. He and Jiang [42] encapsulated isophorone diisocyanate (IPDI) via interfacial polymerization, and polyvinyl alcohol (PVA) was applied as the emulsifier in the oil/water emulsion system. In this field, the stable membrane supports the protection of crucial oil and fragrance compounds against unfavorable degradation and influence caused by the environment, and the selection of the emulsifier is paramount to shell formation.

#### 2.3.2. Coacervation/Phase Separation

**Figure 3:**
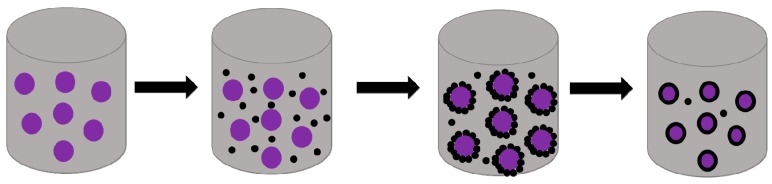
Microencapsulation with coacervation.

Coacervation (or phase separation) is commonly utilized for the preparation of microcapsules enveloped by gelatin [43], gelatin-acacia [44], cellulose derivatives [45], or other synthetic polymers [46,47]. The coacervation is brought about to encapsulate liquids and solids by gradual desolvation of fully solvated polymer molecules (Figure 3). To optimize the process, some additives such as the stabilizer, coacervating agent, and crosslinking agent were added [48,49]. Coacervation is a unique and promising microencapsulation technology, which can achieve high payloads (up to 99%) [29,50]. With a different number of colloidal solutes, the processes are divided into two types, namely simple and complex coacervation. In simple coacervation, single solutes make the process easily controlled. The desolvation of the polymer can be induced by changing the temperature of the polymer solution, by adding a poor solvent or non-solvent for the polymer, or by introducing salts and electrolytes [46,51]. Wang [52] established a new nano encapsulation, in which small nanocapsules with a natural polymeric shell (gelatin) could be fabricated via simple coacervation, capsuling capsaicin that has a pungent odor. In this research, the relative independence to pH gives it priority in some cases, compared with complex coacervation, where the dependence on salt creates another principle for agglomeration, which can be adjusted by the kinetics and the feeding rate of salt in the system, while the complex one is considered to be reduced within the reaction of oppositely charged polyelectrolytes. The most shared couple is gelatin-acacia, and any core materials which can be dispersed in a liquid phase can potentially be enveloped, however, it remains challenging to select materials that do no harm the environment [53,54,55]. Bo [56] prepared gelatin/sodium hexametaphosphate (SHMP) and employed it to encapsulate tuna oil for retarding the oxidation of omega-3 oil, and the cross-linked microcapsules were formed by complex coacervation between the gelatin and SHMP.

#### 2.3.3. Solvent Evaporation

**Figure 4:**
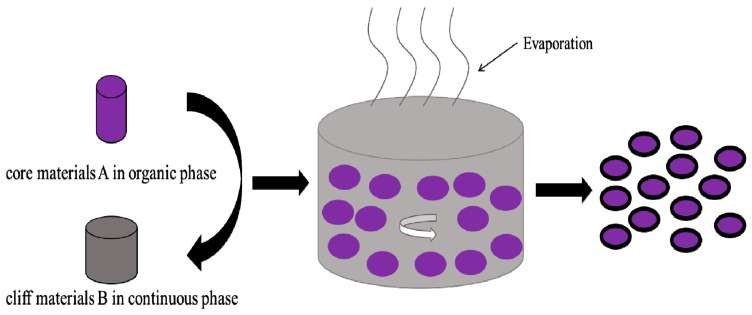
Microencapsulation with solvent evaporation.

Since its development by the end of the 1970s, solvent evaporation has been a promising alternative for microencapsulation, especially for solid core materials, which is very similar to suspension crosslinking, but in this case the polymer is usually a hydrophobic polyester [49]. At first, the coating material is dissolved in a water immiscible volatile solvent, into which the core materials are also dissolved or dispersed. The mixture is then dispersed in the liquid manufacturing vehicle with continuous agitation to obtain tiny capsules with desired size (Figure 4). In the pharmaceutical industries, the obtained polymer microspheres with an active drug trapped inside can degrade and release the encapsulated drug slowly with a specific release profile [57,58,59]. Misal [3] prepared fucoxanthin-loaded microspheres (F-LM) via a two step w/o/w double emulsion solvent evaporation method with poly (l-lactic-co-glycolic acid) (PLGA) as the carrier. Youan [60] encapsulated superoxide dismutase (SOD) in poly(ε-cap- rolactone) (PCL) microparticles by reverse micelle solvent evaporation, and in this way, the SOD bioavailability was improved. This controlled drug release will efficiently reduce dosing frequency, and be more convenient and acceptable for patients, and thus has promising clinical benefits.

### 2.4. The Mechanism of a Microcapsule and Its Development in Oilfield Chemicals

**Figure 5:**
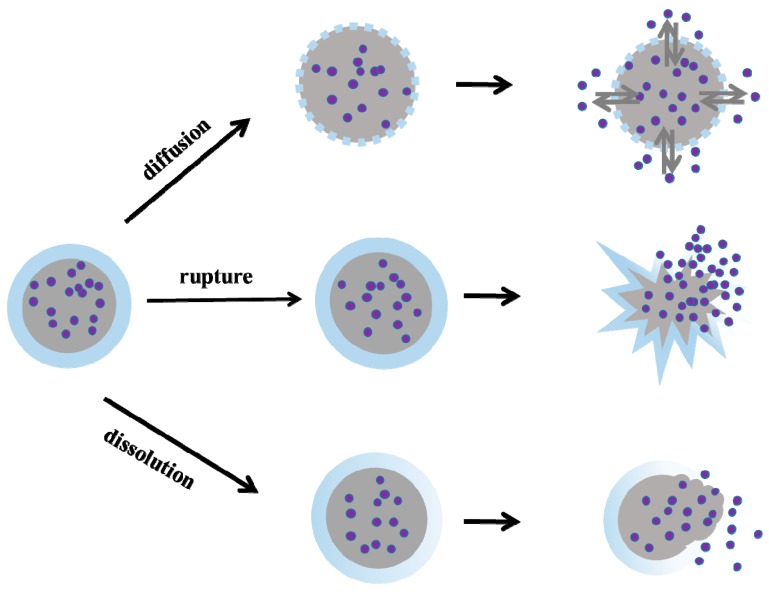
Release mechanism of the core material.

Various mechanisms of microcapsules provide controlled, sustained, and targeted release of the internal phase [49]. Generally, there are three different mechanisms by which the core material is released. More precisely, the action modes of the enclosure membrane, that is, dissolution, rupture, and diffusion, are shown in Figure 5.

**Dissolution.** When the proper temperature is reached, the coating begins to dissolve in water or other non-toxic solvents, and then the active substance inside is gradually released into the external environment to play its role. In the detergent industry, the approach of encapsulated protease enzymes released to remove blood stains and milk stains is due to the dissolution of the powder detergent shell. Food makers utilize this technique to mask taste or keep a flavor from its volatile nature, and when it needs to be released, the membrane will melt with the assistance of the proper temperature [61].

**Rupture.** Rupture of the coating is a commonly shared mechanism, which can be released by pressure or the propagation of cracks. Compared with the dissolution mechanism, the most obvious feature in this process is that these tiny spheres are subject to external forces of extrusion or stretching. Rupture of the outer sheath may be caused by pressure as in the case of carbonless copy paper as well as scratch and sniff perfumes or due to the propagation of cracks as for the repeatedly mentioned self-healing structures [62,63,64], which has been used in concrete [65,66]. Due to the appropriate requirements of the reaction environment, this mechanism provides a potentially promising candidate for use in the petroleum industry, such as deep profile control and crack healing in oil wells. 

**Diffusion.** The utilization of the diffusion mechanism has fundamentally changed the pace of development in the pharmaceutical industry. As a quintessential example, Aspirin provides effective relief from fever, inflammation, and arthritis, but direct doses of aspirin can cause peptic ulcers and bleeding. To diffuse in a slow and sustained dose, aspirin is encapsulated in ethyl cellulose or hydroxypropylmethyl cellulose and starch, and these semipermeable celluloses make it possible for the drug to permeate at the beginning [67,68]. Meanwhile, it is worth noting that some of microcapsules used in oil fields such as delayed crosslinker and controllable retarder, also act in this way. Concentration differences exist between the microcapsule and the environment, thereby allowing the encapsulated materials to diffuse into the environment, where it undergoes irreversible crosslinking to produce super absorbent resin or to conduct cement solidification. Diffusion is hard to control and is easily affected by several factors. In this mechanism, the thickness, hole-rate, and deformation of membrane play a key role to achieve release via diffusion, and for core materials, the solubility, diffusivity, and partition coefficient are paramount.

## 3. Microencapsulation in Oilfield Additives

### 3.1. Current Situation

In the past few decades, the advancement in the development of petroleum industries has led to the emergence of several new industries and contributed to the enhancement of existing ones. Various patents and literatures have been reported to enhance oil recovery, with many focused on engineering exploration. Relevant literatures, which focused on the exploration of chemical assistants, are severely lagging behind. The limited number of papers in this area that we can search is based on field measurement data, which play a minor role in theoretical exploration and structure innovation, due to the incompleteness of the formula and limited applicability of the atmosphere. 

It is hard to believe that this is the current situation regarding this subject, which has flourished for centuries and is still the focus of many nations. There is no doubt that, for any fields, a multitude of theoretical studies will provide energy for its continuous progress, thus in addition to the pursuit of economic efficiency, the improvement and development of theoretical exploration should also be valued. It is hoped that this contribution will play some small part in helping develop future prosperity in this significant field. More crucially, this review is supposed to attract attention to the sustainable development in this field.

### 3.2. Materials

#### 3.2.1. Shell Materials

It is widely understood that additives, when applied in oil recovery, have to have excellent properties regarding salt tolerance, adequate mechanical stability, and temperature resistance [69], to allow for efficiency in the whole process. Microcapsules used in this area probably require a much tighter coating to protect their active cores, while the outer membrane for the rupture mechanism is expected to have the characteristics of easy breakdown under the appropriate conditions [70]. As mentioned above, many types of natural and synthetic polymers are being explored, such as gelatin, chitosan, polyvinyl alcohol, and methylcellulose. All these materials are superior in membrane formability and chemical stability [71].

It is universally acknowledged that the petroleum industry depends largely, almost entirely, on the development of high-tech industrialization, especially for petroleum enterprises in Western countries. Hence, the over reliance on industrialization has relatively left behind the exploration of corresponding chemicals. There is no better example than microcapsules, which were postulated to allow for applications in postponed-swelling, corrosion inhibition, and an array of other applications due to their unique structure. However, due to limited research, the majority of the membranes are still natural materials and conventional polymers. Thus, there is great potential for further development in the preparation and modification of the outer sheath [72].

#### 3.2.2. Core Materials

According to the different functions of the microcapsule in oil recovery, the criterion for the core materials also varies. Herein we will elaborate on the gel breaker, corrosion inhibitor, and self-healing agent [73]. Contrary to the enclosure membrane, it has developed to a relatively advanced level to satisfy the requirements of the petroleum industry.

### 3.3. Preparation and Evaluation Methods

The fabrication of oil field chemicals is preferred to be efficient and convenient. The most commonly used methods, such as interfacial polymerization and coacervation, have been mentioned above. In this section, we mainly focus on the sophisticated measurement and data analysis technologies used to assess additives with microcapsule structures. 

**Measurement.** Transmission electron microscopy (TEM) is the most common method used to explore the structure of the microcapsule, which can prove the coating effect as well as obtain the diameter of the material and the thickness of the membrane. However, the intrinsic drawback of this method is that it cannot visualize structures with a thick shell. Therefore, the morphology is also characterized by scanning electron microscopy (SEM) [74,75], which can finely view the material surface. Components of microcapsules can be investigated by energy dispersive spectroscopy (EDS), X-ray diffraction (XRD), and selected-area electron diffraction (SAED). With the development of analytical techniques, we evaluated the components and morphology at the same time to improve accuracy [76,77]. Other methods, such as differential scanning calorimetry (DSC) and thermogravimetric analysis (TGA), are applied to evaluate the thermo-properties [78,79,80]. Recently, X-ray computed tomography (XCT) scanning technology showed a wondrous ability in evaluating complex or porous systems, and it provides characteristic parameters to form a real system via a related procedure [81]. Lv [82] utilized XCT to observe the status and fracture behavior of microcapsules inside a cement paste matrix, and then elaborated 3D reconstructed tomographic images of the cement matrix with the assistance of 3D rendering based on the segmentation and data from XCT. Novel measurements, such as XCT, surely make it more intuitive to explore the internal structure of materials, and will notably be more conductive for its further development. 

For microcapsules used in the oil field industry, in addition to the nature of the capsule itself, its application performance must be emphasized since all these properties mainly determine its effect in the working process. As an example, salt tolerance and temperature resistance have priority in characterizing all additives used in deep and ultra-deep wells [83,84,85,86]. In addition, some smart microcapsules for healing cracks in the long-term should be characterized by the crack healing ratio or chloride permeability [87] and water permeability [88] to evaluate its self-healing capacity. It is noteworthy that these measurements are more significant than the material properties when considering practical applications.

**Visual characterization.** Visual characterization is particularly significant in the evolution of research under some limiting conditions, such as novel advances, Beyasian, simulation, and modeling. Researchers from North Carolina State University, the National Institute of Standards and Technology, and Oak Ridge National Laboratory found a fantastic method to characterize the structure of materials by using Beyasian data analysis, which will promote the development of analytical techniques [89]. 3D Monte Carlo simulation was utilized in Yio’s research to study the variation of the backscattered electron (BSE) signal across pore-solid boundaries in cement-based materials, where experiments would not be feasible [90]. Recently, in self-healing cement researches, the coupled thermo-hygro-chemical (THC) model was once used to prove that cracks can be healed in ordinary cementitious materials in the presence of water by Chitez [91]. As one of the latest advancements in data analysis, simulation was supposed to be an indispensable addition of conventional experiments and should extend to existing industries.

### 3.4. Main Product and Mechanism

#### 3.4.1. Microencapsulated Delayed Release Breaker

Hydraulic fracturing and fracture acidizing are techniques commonly utilized to stimulate the production of oil and gas from subterranean formations of low permeability. In carrying out such techniques, high viscosity gelled aqueous fluids and water-hydrocarbon emulsions have been utilized as fracturing and fracture-acidizing fluids, which can form the suspension of propping agent without excessive settling, and bring about the opening of one or more fractures in the formation to a greater width. Thus, to recover from the formation through the well bore, the fracturing fluid seeps into the formation or back-flows out from the fractures, and it is desirable to utilize a breaker to convert the gel or emulsion to a low viscosity [92]. As early as 1980, Burnham reported that the preparation of a capsule gel breaker can achieve the purpose of delayed breaking. Since then, Nolte and Walles also reported the successful preparation technology of the capsule gel breaker [93]. Expanding on these studies, Gulbis and his team achieved the encapsulation of a breaker, which was wrapped by a thin film and temporarily shielded from the environment. Moreover, it can be released under the extrusion pressure produced by the crack closure, and thus has the characteristics of delayed release [94]. In 1992, Gulbis and King reported another type of breaking agent with a microcapsule structure. In their research, an enzyme was employed as an internal core, and this product performed effectively in the fracturing formation process at low temperature and pH [95]. Considering the inhibition of low temperature, Wu [96] proposed a new microencapsulated heat-generating hydraulic fracturing fluid system to avoid the low activity of the conventional viscosity breaker at low temperature. Innovation in this field provides an overall understanding of the performance and preparation of the microencapsule gel breaker, and will be further illustrated below.

**Figure 6:**
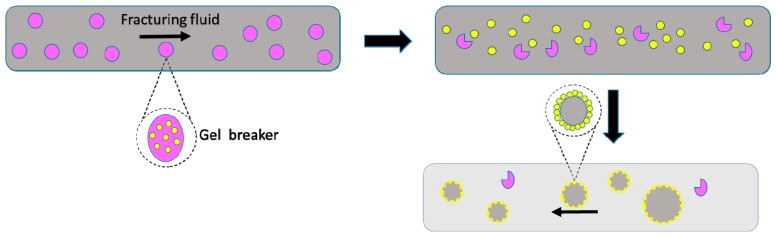
Back-flow process of fracturing fluid.

**Mechanism.** The microencapsulation of breakers achieves the delayed release of active ingredients by coating individual pellets, which allows the breaker to be temporarily isolated from the fracturing fluid. Thus the breaking system tends to release over a desired period of time, for example, a number of hours, days, or weeks, followed by the relatively rapid release of the inner phase, which improves its performance, reduces the residue of the fracturing fluid, and does no harm to the initial viscosity of the fracturing fluid and the sand carrying capacity [92], as shown in Figure 6. To control the release of the microcapsule breaker, we mainly concentrated on the rupture, dissolution, and diffusion of the enclosure membrane, which have been mentioned above. In the working process, the initial exchange in the microcapsule system is inclined towards diffusion, and then it gradually tends to dissolution. While recently the rupture mechanism was found to perform very well in a high-pressure oil well, it is widely known that this mechanism will play a leading role in the gel breaking technique for deep well operation.

**Materials.** Many kinds of chemicals have been utilized to fabricate the special microcapsule structure. The most shared core materials are persulfate oxidizers, such as sodium peroxydisulfate and ammonium peroxydisulfate, which can be used at high temperatures. Considering the environmental degradation that exists in recent years, some enzymes are also applied as core materials in the gel breaker of low pH and low temperature water base fracturing fluid. The enclosure membrane is sufficiently permeable to at least one fluid in the subterranean environment. When injected with the controlled release capsule, the coating is capable of rupturing upon sufficient exposure to the fluid, thereby releasing the internal breaker [97]. The materials of the outer sheath can be cellulose (methylcellulose, ethylcellulose), crosslinked ethylene copolymer (polyvinyl chloride), and gum (guar gum, locust beam gum).

#### 3.4.2. Microencapsulated Oilfield Corrosion Inhibitor

The corrosion of well tubing and operating equipment adversely affects the oil recovery rate, and restricts the exploration of offshore platforms. However, it is arduous to treat the individual wells, which are widely dispersed geographically, and are inaccessible during operation [98,99,100,101,102]. The traditional corrosion inhibitors with their own defects are incapable of solving application problems, such as the adverse influence on the environment and performance instability [101], which will deteriorate the inhibitor efficiency and physical barrier properties in a short period. Microencapsulation of the corrosion inhibitor can extend the treatment over a period of time to avoid the need for frequent operation via controllable release, and decrease unfavorable interactions via protection from the outer sheath. In addition, some researchers have produced anticorrosion coatings with self-healing effects to achieve the maximum corrosion retarding capability and enhanced long-term protection by compensating for the drawbacks of the organic coating system automatically. As an example, Zheludkevic [103] developed of a new protective system with self-healing ability that was composed of hybrid sol-gel films doped with nanocontainers that release entrapped corrosion inhibitors in response to pH changes caused by the corrosion process. Nowadays, studies in this sphere mainly focus on highly efficient, non-toxic, and smart-sensitive microcapsules. Corrosion is a serious issue which has constrained the development of many industries, but with the assistance of core-shell structures, some disadvantages have been overcome. This has tremendous potential for the petroleum industry and provides possibilities for further development of offshore production [104].

**Figure 7:**
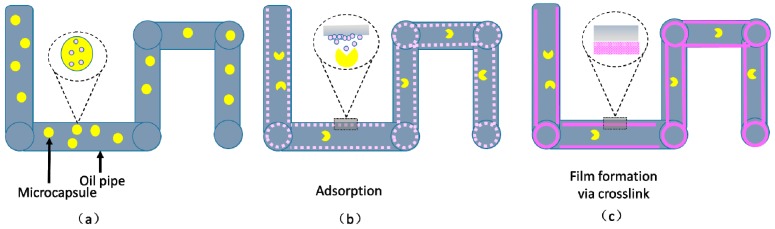
Mechanism of corrosion inhibitor in an oil pipe. (**a**) Transportation of microcapsules; (**b**) release of corrosion inhibitor and (**c**) inert film formation.

**Mechanism.** Microencapsulation of the corrosion inhibitor is a quintessential example of delayed release and physical shielding which was utilized to prolong the anti-corrosion effect and deal with the widely dispersed well tubing. The enclosure membrane provides an isolation system for the corrosion inhibitor to avoid negative influences from the underground environment. To extend the treatment in time and space, diffusion through the micro holes on the surface of the membrane is a potential candidate [101,103,104], which has been explicitly introduced above. After exposure to the environment, the internal phase is supposed to form a passive film on the surface of the metal substrate. To stop the penetration of corrosive factors such as ions, water, and oxygen, the main inhibitive mechanism is considered to be the adsorption on metal surface or complexes with metal substrates [105], followed by the formation of a passive and protective layer, as shown in Figure 7. 

**Materials.** Because of the corrosive environment in oil production, the outer membrane is supposed to be sufficiently permeable to encapsulated liquid, and stable in harsh environments; salt tolerance and high-temperature resistance should be considered [102]. Polyuria [106], Polyurethane (PU) [107], Glutin, and polyvinyl alcohol (PVA) [108] have received considerable attention from many research groups. Thiourea, quinolone, and triethanolamine (TEA) are commonly selected as interphase, which are excellent in metal protection due to good chemical stability and solubility in water, but cannot work well in high sour wells when directly incorporated. Some researchers find that microencapsulation is an effective method to solidify liquid inhibitor components and protect them from harsh conditions; Kuang [108], for example, successfully microencapsulated thiourea (H_2_NCSNH_2_) in PVA and glutin to prolong the anti-corrosion treatment. These materials offer important alternatives for the preparation of smart microcapsule corrosion inhibitors, however further expansion is still attractive.

#### 3.4.3. Microencapsulated Self-Healing Agent

Self-healing is regarded as one of the new technologies in many high-tech fields [37,42], since it was proposed by White in 2001 [109]. It is worth mentioning that this smart system has been applied in oil well cement to repair microannulus produced by trapped pressure and other damages such as planned cycle or operational changes. The cement sheath provides mechanical bonding and supports the casing, and chemically protects the steel casing from corrosion, isolating fluids and gases between the formations. Cracking inside the cement sheath and debonding between the cement sheath and the borehole (or the metal pipe) are unavoidable, which will cause the leakage of oil fluid and will more seriously result in the failure of the whole corrosion system for the steel pipe [110]. Once micro-cracks form in oil well cement, they are difficult to detect and repair by conventional methods, and can only be treated via quadric well cementation and recementing, which is thought to be inefficient and uneconomical. Although some binder materials, such as cellulose [111] and glass fiber [112], were utilized in oil well cement to simply control the initial crack-restraining, they are unable to prevent the late emerging cracks. Self-healing microcapsules provide a novel isolation system for core healing agents during the mechanical mixing process and later curing stage, where a propagating crack appears and the healing agent will flow out to repair it [113]. Up to now, microencapsulation has been widely utilized to improve the service life and stability of construction cement with long-term cracks [87,114,115,116]. Recently, Kim [64] prepared a kind of microcapsule for cementitious materials with secondary crack preventing ability, which marks another breakthrough and will be more rewarding once applied to the cement sheath. Although it represents significant advancements in the development of self-healing, several questions remain due to the differences between building cement and oil well cement. It is still a significant challenge to achieve a leak-free oil well via this novel technology, considering the complex harsh environment, mixing, and curing process. The exposure mechanism and materials of self-healing microcapsules are cited as following.

**Figure 8:**
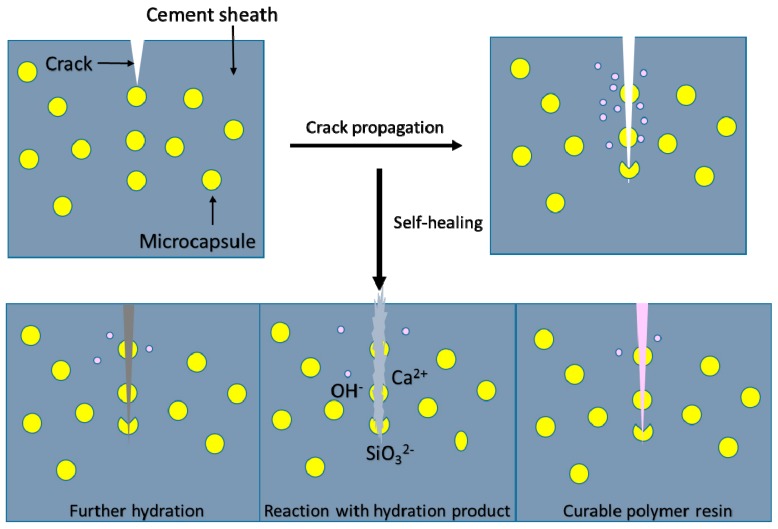
Self-healing mechanism of the cement sheath.

**Mechanism.** Self-healing microcapsules mainly provide a protective isolation system for healing agents to avoid rupture from harsh mixing and curing processes. Smart and sensitive self-healing is realized through the release and reaction of repairing chemicals in the region of damage, when the outer coating is ruptured by cracks. As a trigger mechanism, crack formation responds very fast, which is immediately followed by the release of the healing agent to the reaction (Figure 8). While ensuring that the system works efficiently, good adhesion between the outer part of the capsule and the matrix should be considered [116]. After exposure, the healing capacity mainly depends on the effect of the encapsulated active constituent, which can be summarized as following: (1) Reaction based on the cement components: further hydration and reaction with the hydration products; (2) Introduction of curable polymer resins. 

**Materials.** It is paramount to highlight that the selection for the enclosure membrane in this system is even more challenging, which should be feasible for both mixing and curing processes. Several excellent works have focused on the preparation of self-healing microcapsules; both inorganic (silica gel [116], sodium silicate [117,118], and ceramics [119]) and organic materials (Urea formal-ehyde, polyurethane, and epoxy resin) were utilized as the outer sheath, and some of them demonstrated amazing performance. Wang [120] synthesized new melamine based inert materials, which are flexible under high humidity and become preferentially brittle at low humidity. This coincides with the exact needs for self-healing systems in cement materials, that is, they maintain vitality during the mixing process and are easily broken when cracks appear [121].

However, similar studies are mostly applied in building cement. Besides the disparity in the component content, one of the obvious differences between building cement and oil well cement is the high working temperature. In Yang’s research, the nano-inorganic/organic hybrid shells, composed of methanol modified melamine-formaldehyde resin and nano-particles of calcium carbonate (nano-CaCO_3_) were prepared to be able to survive in the harsh environment of 200 °C [117], which was also promising for oil well cement. Core materials, that is the healing agent, mainly focus on bacteria [122,123,124], inorganic compounds (Na_2_SiO_3_, Na_2_FPO_3_ [125], Ca(OH)_2_ [126]) and crosslinking polymers (epoxy [127], acrylic resin). Ge et al. prepared a new series of microcapsules containing poly(styrene-divinylbenzene) as the shell material and a mixture of epoxy resins as the core material via in-situ polymerization technology [128]. However, this mature technology in construction cement still needs more exploration, when applied in oil well cement for self-healing.

#### 3.4.4. Microencapsulation of Other Additives

Recently microencapsulation has been gradually extended to many traditional additives for the exploration of gas and oil production. As mentioned above, this unique structure can be applied for delayed release to maintain an active effect for a certain period of time. It can also reduce the injection amount of a chemical agent and economize operating costs regarding utilization. The microencapsulation of other agents, such as lost circulation, delayed release crosslinkers, and drag reducing polymers also have captured scholars’ attention. As an example, Chen [129] and his team prepared crosslinking microcapsules for in-depth profile control using CrCl_3_ as the core material encapsulated by polyvinyl alcohol (PVA). With the assistance of SEM and an ultraviolet light photometer, researchers obtained approximately spherical microcapsules, which have obvious delayed release effects. The release time could slow down to 25 h. Another quintessential example is about drag reducing polymers, which are a key factor that controls the pipeline construction, operation cost, and even its security. Pipeline transportation is the main type of transport in commercial applications of transporting oil and natural gas. Thus, Li [130] reported a kind of microcapsule containing α-olefin drag reducing polymer, which could effectively protect α-olefin particles from adhesion, and their contribution is significant to the development of pipeline transportation.

## 4. Prospects for Oilfield Additives with Microcapsule Structures

Studies in the area of microencapsulation have huge potential in giving raw materials advantageous traits, resulting in considerable advancement of existing industries and newly emerging fields, such as biomedicine, food, self-healing materials, and cosmetics. In this review, we emphasized the preparation and mechanisms of microcapsules in some developed fields, and provided an overview of their applications as oilfield additives on a large scale. This microcapsule architecture is superior because it can control the release of active compounds (sustained or delayed release), modify physical characteristics (convert free flowing liquid to solid), and increase stability (protect core materials against degradative reactions such as oxidation). In working processes, the rupture, dissolution, and diffusion effect of the outer sheath provides the possibility for the realization of these functions mentioned above. The fruitful achievements in analytical technique such as the microcapsule structure, and some later measurements and combined techniques make it possible to explore the deeper structure of the microcapsule, for example, XCT combined with 3D rendering, which shows 3D tomographic images of the internal structure of the cement matrix with microcapsules dispersed homogeneously [131,132]. It is not an exaggeration to say that we can open the shell to observe its inner world in the near future. 

Particularly, based on applications in other fields, we introduced microcapsules into the petroleum industry. Improvement in the design, performance, and overall understanding of the microcapsule structure opens up promising avenues for the application of the gel breaker, corrosion inhibitor, and crack-healing, which make full use of the microcapsules’ delayed release and protection effect. With the progress obtained in this field, more demands are imposed. The mature conventional product could never fulfill these requirements. Thus its application in some sophisticated conditions is worth investigating and environmental friendly concepts should be contained in these products. Another limitation in this fields can be concluded as follows; most of our accurate but complex work now in lab-scale setups only offer the theoretical basis for the development of the relative industry, while the manufacturing process requires enough knowledge to scale up to the commercial scale.

As mentioned at the beginning of this paper, petroleum is more than a resource and energy. This high-stake political product has been a symbol of national capability. As chemical researchers focus on petroleum chemicals, additive exploration is the best way in which we can work. The special microcapsule structure, which has been commonly applied in modifying the original product, is commonly designed to achieve functional improvement. Why not try to combine two independent components with various functions in the form of microencapsulation to achieve a multifunctional preparation? Thus the additives’ dose amount and type will be reduced. Additionally, no consideration needs to be given to the cooperation of different chemicals. This hypothesis, along with the ability to control the release and its potential for designing new exciting structures, makes microcapsules promising candidates for intelligent production in the petroleum industry. It is a stimulating challenge and we sincerely hope that this review will play some role in helping future developments in this crucial field.

## References

[1] Obi C.I. (2010). The petroleum industry: A paradox or (sp) oiler of development. J. Contemp. Afr. Stud..

[2] Alagusundaram M., Chetty M.S., Umashankari C. (2009). Microspheres as a novel drug delivery system—A review. Int. J. Chem. Tech. Res..

[3] Misal R., Waghmare A., Aqueel S. (2013). Recent advances in microencapsulation: A review. Int. J. Pharm. Tech..

[4] Sliwka W. (1975). Microencapsulation. Angew. Chem. Int. Ed..

[5] Yang H.I., Kim D.M., Yu H.C., Chung C.M. (2016). Microcapsule-type organogel-based self-healing system having secondary damage preventing capability. ACS Appl. Mater. Interfaces.

[6] Yuan X.Z., Sun W., Zuo X.B. (2011). Study of feasibility of heat melt adhesive being used in crack self-healing of cement-based materials. Appl. Mech. Mater..

[7] Cui H., Tucker-Burden C., Cauffiel S.M.D., Barry A.K., Iwakoshi N.N., Weber C.J., Safley S.A. (2009). Long-term metabolic control of autoimmune diabetes in spontaneously diabetic nonobese diabetic mice by nonvascularized microencapsulated adult porcine islets. Transplantation.

[8] Grandoso L., Ponce S., Manuel I., Arrúec A., Ruiz-Ortegaa J.A., Ulibarria I., Oriveb G., Hernándezb R.M., Rodríguezb A., Rodríguez-Puertasa R. (2007). Long-term survival of encapsulated GDNF secreting cells implanted within the striatum of parkinsonized rats. Int. J. Pharm..

[9] Aoki T., Jin Z., Nishino N., Kato H., Shimizu Y., Niiya T., Murai N., Enami Y., Mitamura K., Koizumi T. (2005). Intrasplenic transplantation of encapsulated hepatocytes decreases mortality and improves liver functions in fulminant hepatic failure from 90% partial hepatectomy in rats. Transplantation.

[10] Wu J., Ding D., Ren G., Xu X., Yin X., Hu Y. (2009). Sustained delivery of endostatin improves the efficacy of therapy in Lewis lung cancer model. J. Control. Release.

[11] Han B., Shen B., Wang Z., Shi M., Li H., Peng C., Zhao Q., Gao C. (2008). Layered microcapsules for daunorubicin loading and release as well as in vitro and in vivo studies. Polym. Adv. Technol..

[12] Kim S.R., Getachew B.A., Park S.J., Kwon O.S., Ryu W.H., Taylor A.D., Bae J., Kim J.H. (2016). Toward microcapsule-embedded self-healing membranes. Environ. Sci. Technol. Lett..

[13] Anal A.K., Singh H. (2007). Recent advances in microencapsulation of probiotics for industrial applications and targeted delivery. Trends Food Sci. Technol..

[14] Saez V., Hernández J.R., Peniche C. (2007). Microspheres as delivery systems for the controlled release of peptides and proteins. Biotecnol. Apl..

[15] Patravale V.B., Mandawgade S.D. (2008). Novel cosmetic delivery systems: An application update. Int. J. Cosmet. Sci..

[16] Leelarasamee N., Howard S.A., Malanga C.J., Ma J.K.H., Castranova V., Ma J.Y.C. (1988). A method for the preparation of polylactic acid microcapsules of controlled particle size and drug loading. J. Microencapsul..

[17] Langdon C.J., De Bevoise A.E. (1990). Effect of microcapsule type on delivery of dietary protein to a marine suspension-feeder, the oyster Crassostrea gigas. Mar. Biol..

[18] Ma J., Liu Y., Bao Y., Liu J., Zhang J. (2013). Research advances in polymer emulsion based on “core-shell” structure particle design. Adv. Colloid Interface Sci..

[19] Han N.X., Xing F.A. (2016). Comprehensive Review of the Study and Development of Microcapsule Based Self-Resilience Systems for Concrete Structures at Shenzhen University. Materials.

[20] Lee D., Park Y., Cho S.H., Yoo M., Jung N., Yun M., Ko W., Jeon S. (2010). Microthermogravimetry of a single microcapsule using silicon microresonators. Anal. Sci..

[21] Ichikawa H., Fukumori Y.A. (2000). Novel positively thermosensitive controlled- release microcapsule with membrane of nano-sized poly(*N*-isopropylacrylamide) gel dispersed in ethylcellulose matrix. J. Control. Release.

[22] Sawada K., Urakawa H. (2005). Preparation of photosensitive color-producing microcapsules utilizing in situ polymerization method. Dyes Pigments.

[23] Jonsson M., Nordin O., Malmström E., Hammer C. (2006). Suspension polymerization of thermally expandable core/shell particles. Polymer.

[24] Kawaguchi Y., Oishi T. (2004). Synthesis and properties of thermoplastic expandable microspheres: The relation between crosslinking density and expandable property. J. Appl. Polym. Sci..

[25] Jeoung S.K., Han I.S., Jung Y.J., Hong S., Shim S.E., Hwang Y.J., Lee P.C., Ha J.U. (2016). Fabrication of thermally expandable core–shell microcapsules using organic and inorganic stabilizers and their application. J. Appl. Polym. Sci..

[26] Posillico E.G. (1986). Microencapsulation Technology for large-scale antibody production. Nat. Biotechnol..

[27] Dubey R., Shami T.C., Rao K.U.B. (2009). Microencapsulation technology and applications. Defence Sci. J..

[28] Dewettinck K., Huyghebaert A. (1999). Fluidized bed coating in food technology. Trends Food Sci. Technol..

[29] Gouin S. (2004). Microencapsulation: Industrial appraisal of existing technologies and trends. Trends Food Sci. Technol..

[30] Knezevic Z., Gosak D., Hraste M., Jalsenjako I. (1998). Fluid-bed microencapsulation of ascorbic acid. J. Microencapsul..

[31] Fukumori Y., Fukuda T., Hanyu Y., Takeuchi Y., Osako Y. (1987). Coating of pharmaceutical powders by fluidized bed process. I. Aqueous enteric coating with methacrylic acid-ethylacrylate copolymer and the dissolution behavior of products. Chem. Pharm. Bull..

[32] Morgan P.W., Kwolek S.L. (1959). Interfacial polycondensation. II. fundamentals of polymer formation at liquid interfaces. J. Polym. Sci..

[33] Schaefgen J.R., Koontz F.H., Tietz R.F. (1959). Interfacial polycondensation. VIII. application to A-B type monomers. J. Polym. Sci..

[34] Chang T.M.S., MacIntosh F.C., Mason S.G. (1966). Semipermeable aqueous microcapsules: I. preparation and properties. Can. J. Physiol. Pharm..

[35] Koishi M., Fukuhara N., Kondo T. (1969). Studies on microcapsules. II. preparation of polyphthalamide microcapsules. Chem. Pharm. Bull..

[36] Yang J., Keller M.W., Moore J.S., White S.R., Sottos N.R. (2008). Microencapsulation of isocyanates for self-healing polymers. Macromolecules.

[37] Lv L., Schlangen E., Yang Z., Xing F. (2016). Micromechanical Properties of a New Polymeric Microcapsule for Self-Healing Cementitious Materials. Materials.

[38] Hirech K., Payan S., Carnelle G., Legrand J. (2003). Microencapsulation of an insecticide by interfacial polymerisation. Powder Technol..

[39] Magdassi S. (1997). Delivery systems in cosmetics. Colloids Surf. A.

[40] Zhang Y., Rochefort D. (2012). Characterisation and applications of microcapsules obtained by interfacial polycondensation. J. Microencapsul..

[41] Mcilroy D.A., Blaiszik B.J., Caruso M.M., White S.R., Moore J.S., Sottos N.R. (2010). Microencapsulation of a reactive liquid-phase amine for self-healing epoxy composites. Macromolecules.

[42] He Z., Jiang S., Li Q., Wang J., Zhao Y., Kang M. (2017). Facile and cost-effective synthesis of isocyanate microcapsules via polyvinyl alcohol-mediated interfacial polymerization and their application in self-healing materials. Compos. Sci. Technol..

[43] Okada J., Kusai A., Ueda S. (1985). Factors affecting microencapsulability in simple gelatin coacervation method. J. Microencapsul..

[44] Kong X.Z., Gu X., Zhu X., Zhang Z. (2009). Spreadable dispersion of insect sex pheromone capsules, preparation via complex coacervation and release control of the encapsulated pheromone component molecule. Biomed. Microdevices.

[45] Weiβ G., Knoch A., Laicher A., Stanislaus F., Daniels R. (1995). Simple coacervation of hydroxypropyl methylcellulose phthalate (HPMCP) II. microencapsulation of ibuprofen. Int. J. Pharm..

[46] Rahimnejad M., Mokhtarian N., Ghasemi M. (2009). Production of protein nanoparticles for food and drug delivery system. Afr. J. Biotechnol..

[47] Niu F., Dong Y., Shen F., Wang J., Liu Y., Su Y., Xu R., Wang J., Yang Y. (2015). Phase separation behavior and structural analysis of ovalbumin–gum arabic complex coacervation. Food Hydrocolloid.

[48] Peanparkdee M., Iwamoto S., Yamauchi R. (2016). Microencapsulation: A review of applications in the food and pharmaceutical industries. Rev. Agric. Sci..

[49] Deshmukh R., Wagh P., Naik J. (2016). Solvent evaporation and spray drying technique for micro-and nanospheres/particles preparation: A review. Dry. Technol..

[50] Yang X., Gao N., Hu L., Li J., Sun Y. (2015). Development and evaluation of novel microcapsules containing poppy-seed oil using complex coacervation. J. Food Eng..

[51] Saravanan M., Rao K.P. (2010). Pectin–gelatin and alginate–gelatin complex coacervation for controlled drug delivery: Influence of anionic polysaccharides and drugs being encapsulated on physicochemical properties of microcapsules. Carbohydr. Polym..

[52] Wang J.C., Chen S.H., Xu Z.C. (2008). Synthesis and properties research on the nanocapsulated capsaicin by simple coacervation method. J. Disper. Sci. Technol..

[53] Butstraen C., Salaün F. (2014). Preparation of microcapsules by complex coacervation of gum Arabic and chitosan. Carbohydr. Polym..

[54] Santos M.G., Bozza F.T., Thomazini M., Favaro-Trindade C.S. (2015). Microencapsulation of xylitol by double emulsion followed by complex coacervation. Food Chem..

[55] Rocha-Selmi G.A., Bozza F.T., Thomazini M., Bolinia H.M.A., Fávaro-Trindadeb C.S. (2013). Microencapsulation of aspartame by double emulsion followed by complex coacervation to provide protection and prolong sweetness. Food Chem..

[56] Bo W., Adhikari B., Barrow C.J. (2014). Optimisation of the microencapsulation of tuna oil in gelatin–sodium hexametaphosphate using complex coacervation. Food Chem..

[57] Li M., Rouaud O., Poncelet D. (2008). Microencapsulation by solvent evaporation: State of the art for process engineering approaches. Int. J. Pharm..

[58] Noviendri D., Jaswir I., Taher M., Mohamed F., Salleh H.M., Noorbatcha I.A., Octavianti F., Lestari W., Hendri R., Ahmad H. (2016). Fabrication of fucoxanthin-loaded microsphere(F-LM) by two steps double-emulsion solvent evaporation method and characterization of fucoxanthin before and after Microencapsulation. J. Oleo Sci..

[59] Manekar N.C., Puranik P.K., Joshi S.B. (1992). Microencapsulation of propranolol hydrochloride by the solvent evaporation technique. J. Microencapsul..

[60] Youan B.B.C. (2003). Microencapsulation of superoxide dismutase into poly(ε- caprolactone) microparticles by reverse micelle solvent evaporation. Drug Deliv..

[61] Wang Y., Lu Z., Wu H., Lv F. (2009). Study on the antibiotic activity of microcapsule curcumin against foodborne pathogens. Int. J. Food Microbiol..

[62] Wu M., Johannesson B., Geiker M. (2012). A review: Self-healing in cementitious materials and engineered cementitious composite as a self-healing material. Constr. Build. Mater..

[63] Li W., Zhu X., Zhao N., Jiang Z. (2016). Preparation and properties of melamine urea-formaldehyde microcapsules for self-healing of cementitious materials. Materials.

[64] Kim D.M., Yu H.C., Yang H.I., Cho Y.J., Lee K.M., Chung C.M. (2017). Microcapsule-Type Self-Healing Protective Coating for Cementitious Composites with Secondary Crack Preventing Ability. Materials.

[65] Dong B., Wang Y., Fang G., Han N., Xing F., Lu Y. (2015). Smart releasing behavior of a chemical self-healing microcapsule in the stimulated concrete pore solution. Cem. Concr. Compos..

[66] Qureshi T.S., Kanellopoulos A., Al-Tabbaa A. (2016). Encapsulation of expansive powder minerals within a concentric glass capsule system for self-healing concrete. Constr. Build. Mater..

[67] Yang C.Y., Tsay S.Y., Tsiang R.C. (2001). Encapsulating aspirin into a surfactant-free ethyl cellulose microsphere using non-toxic solvents by emulsion solvent-evaporation technique. J. Microencapsul..

[68] Pathak Y.V., Shingatgiri M., Dorle A.K. (2008). In vivo performance of pentaestergum-coated aspirin microcapsules. J. Microencapsul..

[69] Horsup D.I., Clark J.C., Binks B.P., Fletcher P.D.I., Hicks J.T. (2010). The fate of oilfield corrosion inhibitors in multiphase systems. Corrosion.

[70] Cantu L.A., Boyd P.A. (2013). Laboratory and field evaluation of a combined fluid-loss-control additive and gel breaker for Fracturing Fluids. SPE Prod. Eng..

[71] Nelson G. (2002). Application of microencapsulation in textiles. Int. J. Pharm..

[72] Cuddington J.T., Moss D.L. (2001). Technological change, depletion, and the U.S. petroleum industry. Am. Econ. Rev..

[73] Heurlin M., Stankevič T., Mickevičius S., Yngman S., Lindgren D., Mikkelsen A., Feidenhans’I R., Borgström M.T., Samuelson L. (2015). Structural properties of wurtzite InP-InGaAs nanowire core–shell hetero structures. Nano Lett..

[74] Tasker A.L., Hitchcock J.P., He L., Baxter E.A., Biggs S., Cayre O.J. (2016). The effect of surfactant chain length on the morphology of poly (methyl methacrylate) microcapsules for fragrance oil encapsulation. J. Colloid Interface Sci..

[75] Krishnamachari P., Hashaikeh R., Tiner M. (2011). Modified cellulose morphologies and its composites: SEM and TEM analysis. Micron.

[76] O'Connell J.H., Lee M.E., Yagoub M.Y.A., Swart H.C., Coetsee E. (2016). Characterization of crystallite morphology for doped strontium fluoride nanophosphors by TEM and XRD. Physica B.

[77] Xu P., Xu J., He M., Song L., Chen D., Guo G., Dai H. (2016). Morphology and chemical characteristics of micro-and Nano-particles in the haze in Beijing studied by XPS and TEM/EDX. Sci. Total Environ..

[78] Abel K.A., Boyer J.C., Andrei C.M., Van Veggel F.C. (2011). Analysis of the shell thickness distribution on NaYF4/NaGdF4 core/shell nanocrystals by EELS and EDS. J. Phys. Chem. Lett..

[79] Zhang K., Zhao Z., Wu Z., Zhou Y. (2015). Synthesis and detection the oxidization of Co cores of Co@ SiO_2_ core-shell nanoparticles by in situ XRD and EXAFS. Nanoscale Res. Lett..

[80] Wong A.B., Brittman S., Yu Y., Dasgupta N.P., Yang P. (2015). Core-shell CdS-Cu_2_S nanorod array solar cells. Nano Lett..

[81] Liu J., Ba M., Du Y., He Z.M., Chen J.B. (2016). Effects of chloride ions on carbonation rate of hardened cement paste by X-ray CT techniques. Constr. Build. Mater..

[82] Lv L., Yang Z., Chen G., Zhu G., Han N., Schlangen E., Xing F. (2016). Synthesis and characterization of a new polymeric microcapsule and feasibility investigation in self-healing cementitious materials. Constr. Build. Mater..

[83] Nalet C., Nonat A. (2016). Retarding effectiveness of hexitols on the hydration of the silicate phases of cement: Interaction with the aluminate and sulfate phases. Cem. Concr. Res..

[84] Nalet C., Nonat A. (2016). Effects of functionality and stereochemistry of small organic molecules on the hydration of tricalcium silicate. Cem. Concr. Res..

[85] Nalet C., Nonat A. (2016). Impacts of hexitols on the hydration of a tricalcium aluminate-calcium sulfate mixture. Cem. Concr. Res..

[86] Nalet C., Nonat A. (2016). Ionic complexation and adsorption of small organic molecules on calcium silicate hydrate: Relation with their retarding effect on the hydration of C_3_S. Cem. Concr. Res..

[87] Dong B., Fang G., Ding W. (2016). Self-healing features in cementitious material with urea–formaldehyde/epoxy microcapsules. Constr. Build. Mater..

[88] Wang J.Y., Soens H., Verstraete W., De Belie N. (2014). Self-healing concrete by use of microencapsulated bacterial spores. Cem. Concr. Res..

[89] Fancher C.M., Han Z., Levin I., Page K., Reich B.J., Smith R.C., Wilson A.G., Jonesa J.L. (2016). Use of bayesian inference in crystallographic structure refinement via full diffraction profile analysis. Sci. Rep..

[90] Yio M.H.N., Wong H.S., Buenfeld N.R. (2016). 3D Monte Carlo simulation of backscattered electron signal variation across pore-solid boundaries in cement-based materials. Cem. Concr. Res..

[91] Chitez A.S., Jefferson A.D. (2016). A coupled thermo-hygro-chemical model for characterising autogenous healing in ordinary cementitious materials. Cem. Concr. Res..

[92] Dai C., Wang K., Liu Y., Li H., Wei Z., Zhao M. (2015). Reutilization of Fracturing Flowback Fluids in Surfactant Flooding for Enhanced Oil Recovery. Energy Fuels.

[93] Nolte K.G. (1985). Fracturing Fluid Breaker System which Is Activated by Fracture Closure. U.S. Patent.

[94] Walles W.E., Williamson T.D., Tomkinson D.L. (1988). Method for Treating Subterranean Formations. U.S. Patent.

[95] Gulbis J., King M.T., Hawkins G.W., Brannon H.D. (1992). Encapsulated breaker for aqueous polymeric fluids. SPE Prod. Eng..

[96] Wu J.Q., Wu X.M., Liu X.J., Zhang N., Liu J. (2005). New hydraulic fracturing fluid with microencapsulated heat-generating system. Oil Drill. Prod. Technol..

[97] Barati R., Johnson S.J., Mccool S., Green D.W., Willhite G.P., Liang J.T. (2011). Fracturing fluid cleanup by controlled release of enzymes from polyelectrolyte complex nanoparticles. J. Appl. Polym. Sci..

[98] Rahimi A., Amiri S. (2015). Anticorrosion hybrid nanocomposite coatings with encapsulated organic corrosion inhibitors. J. Coat. Technol. Res..

[99] Al-Amiery A.A., Kadhum A.A.H., Alobaidy A.H.M., Mohamad A.B., Hoon P.S. (2014). Novel corrosion inhibitor for mild steel in HCl. Materials.

[100] Shi S.C., Su C.C. (2016). Corrosion inhibition of high speed steel by biopolymer HPMC derivatives. Materials.

[101] Zaid G.H., Sanders D.W. (2005). Binary corrosion inhibitors offer improved corrosion control. SPE Prod. Facil..

[102] Thanawala K., Mutneja N., Khanna A.S., Raman R.K. (2014). Development of self-healing coatings based on linseed oil as autonomous repairing agent for corrosion resistance. Materials.

[103] Zheludkevich M.L., Shchukin D.G., Yasakau K.A., Möhwald H., Ferreira M.G. (2007). Anticorrosion coatings with self-healing effect based on nanocontainers impregnated with corrosion inhibitor. Chem. Mater..

[104] Choi H., Song Y.K., Kim K.Y., Park J.M. (2012). Encapsulation of triethanolamine as organic corrosion inhibitor into nanoparticles and its active corrosion protection for steel sheets. Surf. Coat. Technol..

[105] Amar H., Braisaz T., Villemin D., Moreau B. (2008). Thiomorpholin-4-ylmethyl-phosphonic acid and morpholin-4-methyl-phosphonic acid as corrosion inhibitors for carbon steel in natural seawater. Mater. Chem. Phys..

[106] Gite V.V., Tatiya P.D., Marathe R.J., Mahulikar P.P., Hundiwale D.G. (2015). Microencapsulation of quinoline as a corrosion inhibitor in polyurea microcapsules for application in anticorrosive PU coatings. Prog. Org. Coat..

[107] Huang M., Yang J. (2011). Facile microencapsulation of HDI for self-healing anticorrosion coatings. J. Mater. Chem..

[108] Kuang F., Shi T., Wang J., Jia F. (2009). Microencapsulation technology for thiourea corrosion inhibitor. J. Solid State Electrochem..

[109] White S.R., Sottos N.R., Geubelle P.H., Moore J.S., Kessler M.R., Sriram S.R., Brown E.N., Viswanathan S. (2001). Autonomic healing of polymer composites. Nature.

[110] Carey J.W., Wigand M., Chipera S.J., WoldeGabriel G., Pawar R., Lichtnera P.C., Wehner S.C., Raines M.A., Guthrie J.G.D. (2007). Analysis and performance of oil well cement with 30 years of CO_2_ exposure from the SACROC Unit, West Texas, USA. Int. J. Greenh. Gas Control.

[111] Peled A., Jones J., Shah S.P. (2005). Effect of matrix modification on durability of glass fiber reinforced cement composites. Mater. Struct..

[112] Zhou S., Zhu H., Yan Z., Ju J.W., Zhang L. (2016). A micromechanical study of the breakage mechanism of microcapsules in concrete using PFC2D. Constr. Build. Mater..

[113] Kanellopoulos A., Giannaros P., Al-Tabbaa A. (2016). The effect of varying volume fraction of microcapsules on fresh, mechanical and self-healing properties of mortars. Constr. Build. Mater..

[114] Huang H., Ye G. (2012). Simulation of self-healing by further hydration in cementitious materials. Cem. Concr. Compos..

[115] Tittelboom K.V., Belie N.D. (2013). Self-Healing in cementitious materials-a review. Materials.

[116] Wang Y.Y., Su J.F., Schlangen E., Liu Y., Zhang J., Han N., Xing F. (2016). Fabrication and characterization of self-healing microcapsules containing bituminous rejuvenator by a nano-inorganic/organic hybrid method. Constr. Build. Mater..

[117] Yang Z., Hollar J., He X., Shi X. (2011). A self-healing cementitious composite using oil core/silica gel shell microcapsules. Cem. Concr. Compos..

[118] Xu J., Yao W. (2014). Multiscale mechanical quantification of self-healing concrete incorporating non-ureolytic bacteria-based healing agent. Cem. Concr. Res..

[119] Kanellopoulos A., Qureshi T.S., Al-Tabbaa A. (2015). Glass encapsulated minerals for self-healing in cement based composites. Constr. Build. Mater..

[120] Wang J., Van Tittelboom K., De Belie N., Verstraete W. (2012). Use of silica gel or polyurethane immobilized bacteria for self-healing concrete. Constr. Build. Mater..

[121] Van Tittelboom K., De Belie N., Van Loo D., Jacobs P. (2011). Self-healing efficiency of cementitious materials containing tubular capsules filled with healing agent. Cem. Concr. Compos..

[122] García Á., Schlangen E., Van de Ven M., Sierra-Beltrán G. (2010). Preparation of capsules containing rejuvenators for their use in asphalt concrete. J. Hazard. Mater..

[123] Jonkers H.M., Thijssen A., Muyzer G., Copuroglu O., Schlangen E. (2010). Application of bacteria as self-healing agent for the development of sustainable concrete. Ecol. Eng..

[124] Wiktor V., Jonkers H.M. (2011). Quantification of crack-healing in novel bacteria-based self-healing concrete. Cem. Concr. Compos..

[125] Sisomphon K., Copuroglu O., Fraaij A. (2011). Application of encapsulated lightweight aggregate impregnated with sodium monofluorophosphate as a self-healing agent in blast furnace slag mortar. Heron.

[126] Huang H., Ye G. (2014). Self-healing of cracks in cement paste affected by additional Ca^2+^ ions in the healing agent. J. Intell. Mater. Syst. Struct..

[127] Liu B., Ke J.L., Deng X., Zhu G.M., Luo Y.C., Han N.X., Xing F. A waterproof epoxy resin microcapsule for the encapsulation of self-healing bacterium. Proceedings of the Fifth International Conference on Self-Healing Materials.

[128] Ge S., Wan F., Lu J., Yu J. (2011). Molecular self-Assembled microcapsules prepared by in situ polymerization technology for self-Healing cement materials. J. Inorg. Organomet. Polym. Mater..

[129] Chen X., Dai J., Zhou M., Hu C. (2014). Preparation of crosslinker microcapsules for in-depth profile control and study on the release model. Spéc. Petrochem..

[130] Li B., Dong G., Zhang C. (2011). Storage stability and solubility of poly (urea-formaldehyde) microcapsules containing A-olefin drag reducing polymer. J. Appl. Polym. Sci..

[131] Schock J., Liebl S., Achterhold K., Pfeiffer F. (2016). Obtaining the spacing factor of microporous concrete using high-resolution dual energy X-ray micro CT. Cem. Concr. Res..

[132] Yang L., Zhang Y., Liu Z., Zhao P., Liu C. (2015). In-situ tracking of water transport in cement paste using X-ray computed tomography combined with CsCl enhancing. Mater. Lett..

